# Supplementary material for: The impacts of bovine milk, soy beverage, or almond beverage on the growing rat microbiome
Source: PeerJ. 2022 May 10;10:e13415. doi: 10.7717/peerj.13415 (PMC9104089; doi:10.7717/peerj.13415)
Supplement: Supplemental Information 2 [file peerj-10-13415-s002.docx]

**Supplementary Figures and tables**

**The impacts of bovine milk, soy beverage, or almond beverage on the growing rat microbiome**

Julie Cakebread^1, 2^, Olivia A. M. Wallace^1^, Harold Henderson^1^, Ruy Jauregui^3^, Wayne Young^2^, Alison Hodgkinson^1^

1. Food and Biobased products, AgResearch, Ruakura Research Centre, 10 Bisley Road, Hamilton, 3240, New Zealand
2. Smart Foods Innovation Centre of Excellence, AgResearch Ltd, Private Bag 11 008, Palmerston North 4442, New Zealand
3. Digital Agriculture Innovation Centre of Excellence, AgResearch Ltd, Private Bag 11 008, Palmerston North 4442, New Zealand

Corresponding author: Julie A Cakebread

Postal address: AgResearch, Private Bag 11008, Manawatu Mail Centre, Palmerston North, 4442, New Zealand.

Email address: [julie.cakebread@agresearch.co.nz](mailto:julie.cakebread@agresearch.co.nz)

# Figure S1 Primers for Illumina MiSeq sequencing:

Forward primer: AATGATACGGCGACCACCGAGATCTACACxxxxxxxxTATGGTAATTGG**CCTACGGGAGGCAGCAG**

where xxxxxxxx is the barcode sequence and CCTACGGGAGGCAGCAG is the 16SV3 forward primer sequence

Reverse primer: CAAGCAGAAGACGGCATACGAGATxxxxxxxxAGTCAGTCAGCC**GGACTACHVGGGTWTCTAAT**

Where xxxxxxxx is the barcode sequence and GGACTACHVGGGTWTCTAAT is the 16SV4 reverse primer sequence.

# Supplementary Table S1 Composition of the AIN-93G food^1^

| Food | Casein | AA-Casein |
| --- | --- | --- |
| **Ingredient** | **gm** | **gm** |
| **Casein** | 200 | 0 |
|  |  |  |
| L-Alanine | 0 | 4.9 |
| L-Arginine | 0 | 6.3 |
| L-Aspartic Acid | 0 | 11.9 |
| L-Cystine | 3 | 4 |
| L-Glutamic Acid | 0 | 36.8 |
| Glycine | 0 | 3.1 |
| L-Histidine | 0 | 4.6 |
| L-Isoleucine | 0 | 8.6 |
| L-Leucine | 0 | 15.3 |
| L-Lysine, HCl | 0 | 13.3 |
| L-Methioinine | 0 | 5.0 |
| L-Phenylalanine | 0 | 8.5 |
| L-Proline | 0 | 17.7 |
| L-Serine | 0 | 9.6 |
| L-Threonine | 0 | 7.0 |
| L-Tryptophan | 0 | 2.1 |
| L-Tyrosine | 0 | 9.6 |
| L-Valine | 0 | 10.7 |
|  |  |  |
| Corn Starch | 397.486 | 397.486 |
| Maltodextrin 10 | 132 | 132 |
| Sucrose | 107.0777 | 107.0777 |
|  |  |  |
| Cellulose, BW200 | 50 | 50 |
|  |  |  |
| Soybean Oil | 70 | 70 |
| t-BHQ | 0.014 | 0.014 |
|  |  |  |
| ^1^Mineral Mix S10022C | 3.5 | 3.5 |
| Sodium Chloride | 7.5 | 7.6 |
| Calcium Carbonate | 12.495 | 3.2 |
| Calcium Phosphate, Dibasic | 0 | 12.5 |
| Potassium Phosphate, Mono | 6.86 | 0 |
| Potassium Citrate, 1 H2O | 2.4773 | 8 |
|  |  |  |
| ^1^Vitamin Mix V10037 | 10 | 10 |
| Choline Bitartrate | 2.5 | 2.5 |
| Biotin, 1% | 0 | 0 |
|  |  |  |
|  |  |  |
| **Total** | **1004.91** | **982.88** |
|  |  |  |
|  |  |  |
| Food (cont) | Casein | AA-Casein |
| **gm** |  |  |
| Protein | 179.0 | 179.0 |
| Carbohydrate | 646.6 | 646.6 |
| Fat | 70.0 | 70.0 |
| Calcium | 5.0 | 5.0 |
| Phosphorus | 2.9 | 2.9 |
| Potassium | 3.6 | 3.6 |
| Sodium | 3.0 | 3.0 |
|  |  |  |
| **gm%** |  |  |
| Protein | 17.9 | 17.9 |
| Carbohydrate | 64.7 | 64.7 |
| Fat | 7.0 | 7.0 |
|  |  |  |
| **kcal** |  |  |
| Protein | 716.0 | 716.0 |
| Carbohydrate | 2586.3 | 2586.3 |
| Fat | 630.0 | 630.0 |
| Total | **3932.3** | **3932.3** |
|  |  |  |
| **kcal%** |  |  |
| Protein | 18.2 | 18.2 |
| Carbohydrate | 65.8 | 65.8 |
| Fat | 16.0 | 16.0 |
| Total | **100.0** | **100.0** |

^1^Standard AIN-93G rodent chow containing casein protein (CN; 20%; Reeves, P. G.Nielsen, F. H.

Fahey, G. C., Jr. (1993) AIN-93 purified diets for laboratory rodents: final report of the American Institute of Nutrition ad hoc writing committee on the reformulation of the AIN-76A rodent diet. J Nutr 123: 1939-51) and a modified version of AIN-93G chow containing amino acids (AA) equivalent to CN, were purchased from Research Diets Inc., (New Jersey, USA).

# Supplementary Table S2 Composition of liquids used as supplements in the trial

| Component | Beverage | | |
| --- | --- | --- | --- |
|  | Milk | Soy | Almond |
| Energy (kcal/100mL) | 64.8 | 65.5 | 28.6 |
| Fat % w/w | 3.49 | 3.3 | 1.79 |
| Protein (%) w/w Kjeldahl | 3.54 | 3.08 | 0.79 |
| ^1^Carbohydrates | 4.70 | 5.10 | 3.30 |
| Total solids (%) w/w | 12.31 | 12.05 | 5.36 |
| Non casein nitrogen (%) w/w Kjeldahl | 0.04 | 0.03 | 0.05 |
| Non protein nitrogen (%) w/w Kjeldahl | 0.03 | 0.02 | 0.03 |
| Phosphate (mg/100mL) | 116 | 158 | 13 |
| Calcium (mg/100mL) | 127 | 181 | 105 |
| Sodium (mg/100mL) | 37.4 | 39.3 | 41.9 |
| Potassium (mg/100mL) | 154 | 263 | 25.7 |
| Magnesium (mg/100mL) | 11.9 | 15 | 5.11 |

1. Carbohydrate content as listed on carton.

Fibre was not measured and was not listed on the cartons.

All other components were analysed by Milk Test NZ and Assure Quality, Hamilton, New Zealand.

# Supplementary Table S3 Starting and final weights of animals and total calorie (kcal) intake for each group

Intake by group of food (g) or liquid (mL) was recorded during the study. Diet and liquid macronutrients were calculated from recorded intake and composition data in tables S1 and S2.

Weights at the start of the trial were not significantly different (average 47.5g, range 34.2g - 61.6 g) At the end of the trial weights were analysed using Genstat (weight day 0 as covariate). Values are shown as means, standard errors of difference (s.e.d) 10.91, n = 10 rats/group. Dissimilar letters signify significant difference.

Supplementary Table S4 Relative abundance (Phyla)

Almost all phyla (10/11) were significantly different (FDR<0.05) in relative abundance. Mean percent ± SEM. Dissimilar letters indicate groups that are significantly different.

Supplementary Table S5 **Statistical analysis of all diets using Permutation ANOVA (Family)**

*40* family level taxa were significantly different (FDR<0.05) in relative abundance (here the top 12 based on highest proportions are shown. Mean percent ± SEM. Dissimilar letters indicate groups that are significantly different.
